# Growth hormone and nonalcoholic fatty liver disease

**DOI:** 10.1097/IN9.0000000000000030

**Published:** 2023-07-27

**Authors:** Ingrid L. Ma, Takara L. Stanley

**Affiliations:** 1 Metabolism Unit, Endocrine Division, Massachusetts General Hospital, Boston, MA, USA; 2 Pediatric Endocrine Division, Massachusetts General Hospital, Boston, MA, USA

**Keywords:** growth hormone, nonalcoholic fatty liver disease, insulin-like growth factor-1, nonalcoholic steatohepatitis, growth hormone, releasing hormone

## Abstract

Nonalcoholic fatty liver disease (NAFLD) is a prevalent cause of liver disease and metabolic comorbidities. Obesity is strongly associated with NAFLD and is also a state of relative deficiency of growth hormone (GH). Evidence supports a role of reduced GH and insulin-like growth factor-1 (IGF-1) in NAFLD pathogenesis. Physiological actions of GH in the liver include suppression of de novo lipogenesis (DNL) and promotion of lipid beta-oxidation, and GH also appears to have anti-inflammatory actions. Physiologic actions of IGF-1 include suppression of inflammatory and fibrogenic pathways important in the evolution from steatosis to steatohepatitis and fibrosis. Rodent models of impaired hepatic GH signaling show the development of steatosis, sometimes accompanied by inflammation, hepatocellular damage, and fibrosis, and these changes are ameliorated by treatment with GH and/or IGF-1. In humans, individuals with GH deficiency and GH resistance demonstrate an increased prevalence of NAFLD compared to controls, with improvement in hepatic lipid, steatohepatitis, and fibrosis following GH replacement. As a corollary, individuals with GH excess demonstrate lower hepatic lipid compared to controls along with increased hepatic lipid following treatment to normalize GH levels. Clinical trials demonstrate that augmentation of GH reduces hepatic lipid content in individuals with NAFLD and may also ameliorate steatohepatitis and fibrosis. Taken together, evidence supports an important role for perturbations in the GH/IGF-1 axis as one of the pathogenic mechanisms of NAFLD and suggests that further study is needed to assess whether augmentation of GH and/or IGF-1 may be a safe and effective therapeutic strategy for NAFLD.

## 1. Introduction

Nonalcoholic fatty liver disease (NAFLD) is a spectrum of disease with the common characteristic of steatosis, which is excessive accumulation of lipid within hepatocytes. The majority of individuals with “simple steatosis”, defined as lipid accumulation without other associated features of disease, will not experience any hepatic impairment and will not progress to further disease stages. In a minority, however, lipid accumulation within hepatocytes will be accompanied by inflammation and hepatocellular damage, which characterize nonalcoholic steatohepatitis (NASH). NASH may progress to fibrosis, cirrhosis, hepatocellular carcinoma, and liver failure. NAFLD is a growing public health concern ^[1,2]^. It is estimated that the prevalence of NAFLD in the United States is 25% to 38% in adults, with comparable prevalence in Europe and Asia ^[3,4]^. NAFLD is more prevalent in individuals with obesity and metabolic dysfunction, and it is estimated to occur in 55% to 70% of those with type 2 diabetes ^[5]^. In children, the prevalence is estimated at approximately 7% to 8% in the general population and 30% to 35% in children with obesity ^[6]^. Further, current trends suggest that NAFLD will replace Hepatitis C as the leading cause of liver transplant by 2030 ^[7]^.

NAFLD is defined as evidence of hepatic steatosis either by imaging or biopsy, along with lack of other known causes of steatosis including significant alcohol use ^[8]^. Steatosis can be quantified by the gold standard of liver biopsy or by using various magnetic resonance imaging (MRI) or spectroscopy (MRS) techniques. Given the invasiveness of liver biopsy, imaging is highly utilized to diagnose NAFLD, and discovery of better biomarkers for NASH and fibrosis are greatly needed. Using MRI or MRS, NAFLD is defined as having ≥5% hepatic steatosis ^[3,8]^. NAFLD is increasingly recognized as a metabolic disease. A bidirectional relationship exists between NAFLD and metabolic comorbidities including obesity, type II diabetes, hyperlipidemia, hypertension, and metabolic syndrome ^[3]^, such that having other metabolic comorbidities increases the risk of developing NAFLD, and having NAFLD increases the risk of developing other comorbidities in this cluster.

Although individuals across the spectrum of body weight may develop NAFLD, there is a strong association between NAFLD and obesity, such that the majority of individuals with NAFLD also have obesity. Importantly, individuals with obesity also demonstrate relative reductions in GH secretory capacity ^[9–11]^. Reductions in GH are most strongly associated with increased abdominal adiposity. Although the exact etiology of GH reduction is unclear, increased circulating free fatty acids (FFA) and hyperinsulinemia are thought to be important mechanisms ^[12,13]^.

New therapeutic strategies to treat NAFLD are urgently needed. Currently, the therapy for NAFLD is weight reduction, which successfully reduces liver fat and biomarkers of hepatic injury such as alanine aminotransferase (ALT) and aspartate aminotransferase (AST) ^[14]^. Increased physical activity has an independent effect to reduce hepatic steatosis ^[15]^, presumably through mechanisms including increased insulin sensitivity, reduction in de novo lipogenesis (DNL), and decreased FFA flux to the liver ^[16]^. Lifestyle modifications are challenging for some patients, however, and maintaining weight loss is difficult ^[17]^. Although multiple mechanistic pathways are being investigated as targets for pharmacological therapy, currently, there is no pharmacologic treatment with specific regulatory approval for NAFLD or NASH ^[2]^. Recent research has highlighted the role of perturbations in the GH axis in the pathogenesis of NAFLD. The roles of GH and insulin-like growth factor-1 (IGF-1) in the liver are described in detail below, along with the evidence base linking perturbations in GH/IGF-1 to the development and progression of NAFLD.

## 2. Overview of the GH/IGF-1 axis

**Figure 1.**
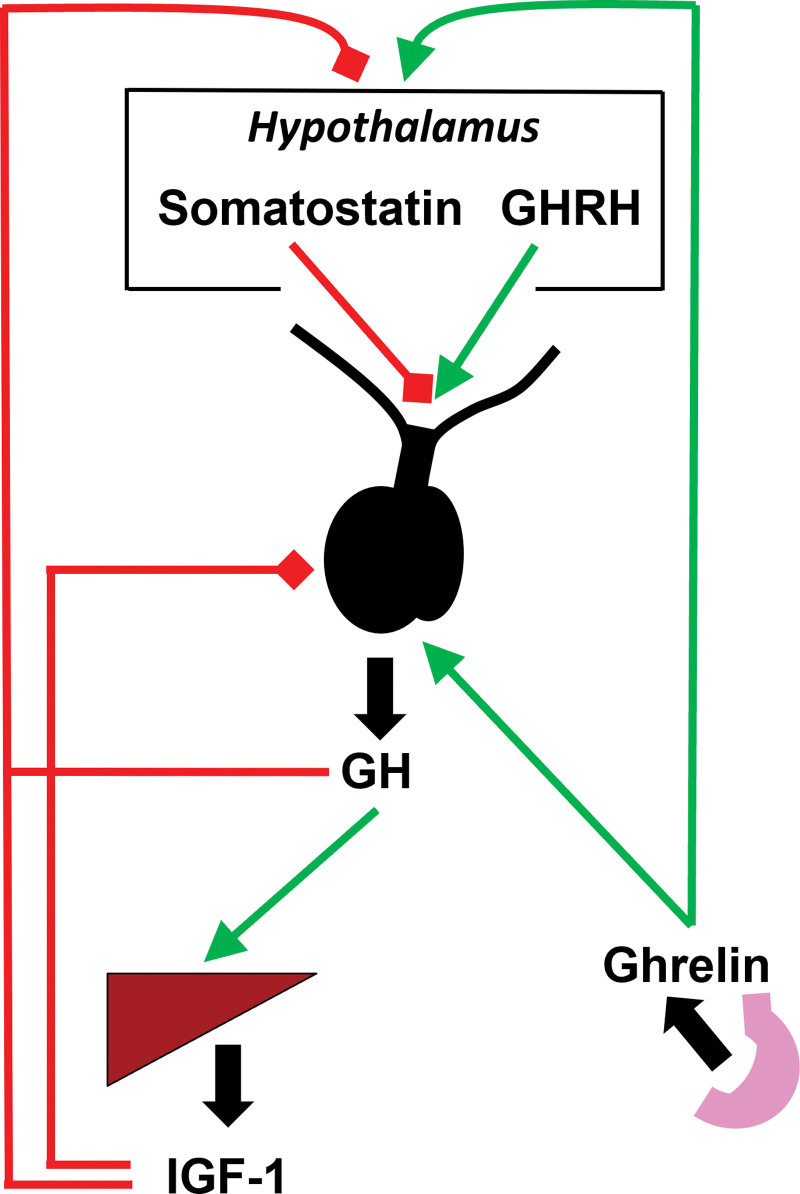
**Regulation of GH secretion.** GH is synthesized and secreted by the anterior pituitary. Pulsatile secretion is regulated primarily by two hypothalamic hormones: somatostatin, which inhibits GH secretion, and GHRH, which stimulates GH secretion. GH negatively regulates its own secretion through hypothalamic signaling, and IGF-1, synthesized in response to GH signaling, also exerts negative feedback at the level of the pituitary and the hypothalamus. Ghrelin stimulates GH secretion at the anterior pituitary and hypothalamus. GH: growth hormone, GHRH: growth hormone releasing hormone, IGF-1: insulin-like growth factor-1.

The pituitary gland synthesizes and secretes GH under the positive regulation of GH-releasing hormone (GHRH) and the negative regulation of somatostatin (Figure 1), both of which are hypothalamic hormones. GH signaling in the liver leads to hepatocyte production of IGF-1, and a negative feedback loop exists such that IGF-1 signals in the hypothalamus and anterior pituitary to decrease secretion of GH ^[18,19]^. Additionally, GH negatively regulates its own secretion through signaling in the hypothalamus ^[20]^. Ghrelin, produced and secreted predominantly from the stomach and also from the hypothalamus, acts at the GH secretagogue receptor (GHSR) in the pituitary and hypothalamus to increase GH secretion ^[21]^. In addition to these primary mediators of GH secretion, numerous other stimuli, including sleep, exercise, and hypoglycemia, increase GH secretion, whereas other inhibitors of GH secretion include free fatty acids (FFA), hyperinsulinemia, and hyperglycemia ^[12]^.

GH secretion is pulsatile, and GH receptors (GHRs) are present in nearly all cell types except neurons, including in multiple cell types in liver, muscle, bone, adipose tissue, heart, and kidney. In the liver, GH signaling occurs in hepatocytes, Kupffer cells, and endothelial cells ^[22]^. GH binding to the homodimeric GHR activates the Janus kinase 2 (JAK2) tyrosine kinase, which initiates signaling through downstream pathways including activation of signal transducer and activator of transcription 5A (STAT5A) and STAT5B, STAT3, and STAT1 ^[23,24]^. Hepatic GH signaling occurs primarily through phosphorylation and nuclear translocation of STAT5B, and, to a lesser degree, STAT5A, collectively referred to here as STAT5 ^[24,25]^. With hepatocyte deletion of STAT5, compensatory signaling occurs through STAT3 ^[22]^. Hepatic STAT5 activation results in transcription of *Igf1*, acid labile subunit (*Als*, which binds to IGF-1 in circulation), and suppressor of cytokine signaling 2 (*Socs2*) ^[24]^. Both IGF-1 and SOCS2 exert negative feedback on further GH-induced gene expression ^[24]^. The glucocorticoid receptor (GR) is an important cofactor for STAT5 ^[24,26]^, and simultaneous impairment of hepatic GHR and GR signaling results in a more severe hepatic phenotype than deletion of GHR alone ^[26]^.

GH is integral to normal growth in childhood ^[27]^. Additionally, throughout the body and into adulthood, GH functions to regulate metabolism, serving as a counterregulatory hormone that stimulates lipolysis and antagonizes insulin action, thereby increasing serum glucose. GH also plays an integral role in regulating body composition and bone health ^[27]^. IGF-1 has independent anabolic effects on skeletal muscle and bone ^[27,28]^ and exerts an insulin-like action that lowers serum glucose.

## 3. Effects of GH and IGF-1 in the liver

**Figure 2.**
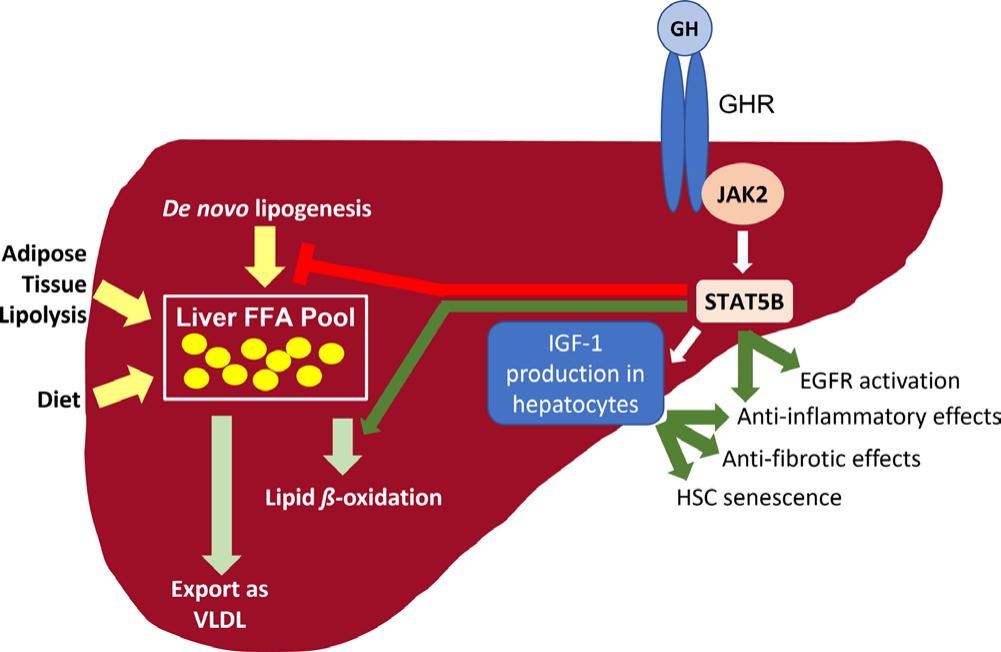
**Effects of GH and IGF-1 on NAFLD and NASH.** Lipid flux to the liver includes FFA from adipose tissue lipolysis, dietary sources, and DNL. Sources of “outflow” from the hepatic FFA pool include synthesis of triglyceride and export as VLDL as well as lipid beta-oxidation. GH suppresses DNL and increases lipid beta-oxidation. GH also increases epidermal growth factor receptor, leading to liver regeneration, and exerts anti-inflammatory effects in the liver. IGF-1 also signals in immune cells in the liver to exert anti-inflammatory and anti-fibrotic effects and to induce HSC senescence. DNL: de novo lipogenesis, EGFR: epidermal growth factor receptor, FFA: free fatty acid, GH: growth hormone, GHR: growth hormone receptor, HSC: hepatic stellate cell, IGF-1: insulin-like growth factor-1, JAK2: Janus kinase 2, STAT5b: signal transducer and activator of transcription 5, VLDL: very-low-density lipoprotein.

GH and IGF-1 act on multiple pathways relevant to the development and progression of NAFLD and NASH (Figure 2). GH increases lipolysis in adipose tissue, mobilizing FFA into systemic circulation ^[29]^. GH also increases the beta-oxidation of FFA ^[30–32]^, an effect expected to decrease hepatic lipid. Additionally, GH suppresses hepatic DNL ^[33–38]^, a process that is an important contributor to stored hepatic lipid in NAFLD ^[39]^. In contrast, impairment of GH signaling increases expression of peroxisome proliferator-activated receptor gamma (*Pparg,* encoding PPARɣ that activates pathways involved in fatty acid uptake and lipogenesis) and cluster of differentiation 36 (*Cd36,* encoding CD36 that binds FFA and facilitates their hepatic uptake) ^[24]^. GH signaling also plays a key role in liver regeneration. GH receptor knockout models have demonstrated that elimination of GH signaling following partial hepatectomy reduces the synthesis and activation of the epidermal growth factor receptor necessary for hepatocyte regeneration ^[40]^ and leads to excessive inflammation and increased apoptosis ^[41]^. Finally, STAT5 exerts an important tumor suppressor effect in the liver, with loss of STAT5 in hepatocytes and mouse embryonic fibroblasts leading to enhanced cell proliferation ^[42,43]^.

Evidence suggests that IGF-1 exerts independent anti-inflammatory and anti-fibrotic effects in the liver. Although normal hepatocytes do not express the IGF-1 receptor (IGF-1R), hepatic non-parenchymal cells do express IGF-1R ^[28,44]^, and IGF-1 affects multiple pathways relevant to NASH pathogenesis. IGF-1 has an anti-inflammatory effect in human hepatoma cell lines, reducing c-reactive protein (CRP) and fibrinogen mRNA transcripts ^[45]^. In models of cirrhosis, IGF-1 exerts anti-fibrotic effects that include reduced activation of hepatic stellate cells (HSCs), upregulation of matrix metalloproteases (MMPs), downregulation of tissue inhibitors of MMPs (TIM-1 and TIM-2), downregulation of profibrogenic molecules including transforming growth factor beta-1 (TGFß1), and induction of cytoprotective factors including hepatocyte growth factor (HGF) ^[44,46,47]^. Similarly, in a NASH model, IGF-1 induces HSC senescence ^[48]^. It is important to note that patients with malnutrition and/or chronic liver disease demonstrate GH resistance, with decreased levels of serum IGF-1 despite regular or elevated GH secretion ^[27]^. Additional effects of GH and IGF-1 on hepatic immune activation and inflammation are elucidated by the animal and human studies discussed below.

## 4. Rodent models: hepatic effects of impaired GH signaling

Various rodent models, shown in **Table 1**, highlight the importance of GH and IGF-1 in hepatic metabolism and inflammatory milieu. Before discussing each model, we highlight two points that are critical in interpreting results and understanding apparent discrepancies between models. First, rodents demonstrate sexual dimorphism in GH secretion to a degree that is not present in humans. GH secretion in male mice and rats is more pulsatile compared with GH secretion in females, who demonstrate lower GH pulse-amplitude and higher inter-pulse GH concentrations ^[57]^. Second, most of the presented models describe *liver-specific* impairment of GH signaling, which has important systemic effects that affect hepatic phenotype. When hepatic GH signaling is impaired or absent, circulating IGF-1 is dramatically reduced. Low circulating IGF-1, in turn, results in high circulating GH, because the usual negative feedback exerted by IGF-1 at the pituitary and hypothalamus is diminished. For models in which whole-body GH signaling is impaired, increased circulating GH does not exert effects. For models in which GH signaling is impaired only in the liver, however, high circulating GH substantially increases adipose tissue lipolysis, raising circulating FFA and further promoting the development of fatty liver. This distinction likely explains why Laron dwarf mice, with systemic GH receptor inactivity, do not have fatty liver, whereas models of liver-specific GH receptor knockout generally do ^[50,55,56,58]^. Nordstrom and colleagues demonstrate this important principle, showing that adipocyte-specific deletion of JAK2 almost completely reverses the NAFLD phenotype in mice with hepatocyte-specific deletion of JAK2, proving that GH-stimulated adipocyte lipolysis is necessary for the steatotic phenotype seen in mice with liver-specific GH signaling defects ^[59]^.

**Table 1.** Liver phenotype in rodent models of impaired GH signaling.

Model, year published	Circulating GH and IGF-1	Hepatic phenotype	Systemic phenotype	Effects of GH and/or IGF-1 replacement/modulation
Hepatocyte-specific deletion of *Stat5,* 2007 ^[49]^	↑↑ GH ↓ IGF-1	**Steatosis**	↑Systemic IR, ↑ glucose	(N/A)
Liver-specific GHR deletion, 2009 ^[50]^	4-fold ↑ GH >90% ↓ IGF-1	**Steatosis** (↑ DNL, ↓ hepatic TG secretion)	↑Systemic IR, ↑ circulating FFA	Rescue of GHR restored glucose tolerance and hepatic TG secretion; IGF-1 infusion normalized serum GH but did not correct hepatic steatosis
Hepatocyte-specific deletion of JAK2, 2011 ^[51]^	↑GH 227% (M)/1040%(F) >90% ↓ IGF-1	**Steatosis** (20-fold ↑TG content), **mild lobular inflammation** (mild ↑ALT, AST), fibroplasia	Reduced weight and fat mass, ↑ circulating FFA	Abrogation of GH signaling by crossing mice with liver-specific JAK2 deletion and *little* mice (with GHRH mutation resulting in inability to secrete GH) rescues fatty liver phenotype
Systemic GHR mutations impairing or eliminating ability to activate STAT5, 2011 ^[52]^	(Not reported)	**Steatosis** (upregulated genes relating to lipid uptake, synthesis, and transport), **inflammation** (↑ALT, AST), **hepatocyte injury** (hepatocyte ballooning)	(Not reported)	(N/A)
Hepatocyte-specific deletion of JAK2 fed high fat diet (HFD), 2012 ^[53]^	↑ GH ↓ IGF-1	**Steatosis** within 2-3 months of age, did **not** progress to steatohepatitis on HFD	Reduced weight and fat mass, ↑ energy expenditure, reduced hepatic insulin sensitivity but normal systemic insulin sensitivity	(N/A)
Spontaneous dwarf rat (mutation in GH gene), 2012 ^[54]^	Absent circulating GH, ↓↓ IGF-1	**Steatosis** (impaired beta-oxidation, **hepatocyte injury** (↑ ALT, AST; smaller mitochondria with altered shape; enhanced oxidative stress), **fibrosis**	(Only liver phenotype comprehensively reported)	Administration of GH (which also restored IGF-1 levels) reversed steatosis, hepatocyte injury, and fibrosis. Administration of IGF-1 to restore serum IGF-1 but not GH also reversed these three features.
Liver-specific GHR deletion, 2014 ^[55]^	~300% ↑ GH ~90% ↓ IGF-1	**Steatosis** in male mice but not female mice	Reduced length, weight, fat mass in adulthood; ↑ glucose	(N/A)
Liver-specific GHR deletion, 2016 ^[56]^	~5-fold ↑ GH ~95% ↓ IGF-1	**Steatosis** (6x increase in hepatic TG; ↑DNL, ↑hepatic lipid uptake); ↑**inflammation** (↑ ALT, AST, markers of oxidative stress, ↑inflammatory cytokines)	↑Systemic IR, ↑ glucose; ↑TG, ↑FFA, ↑cholesterol	Restoration of hepatic IGF-1 expression via transgene (1) normalized serum GH, glucose homeostasis, and serum lipid; (2) decreased but did not normalize hepatic steatosis, DNL, or hepatic lipid uptake; (3) did not affect enzymes involved in oxidative stress or inflammatory cytokines but did normalize ALT, AST, and lipid peroxidation

ALT: alanine aminotransferase, AST: aspartate aminotransferase, DNL: de novo lipogenesis, F: females, GH: growth hormone, GHR: growth hormone receptor, FFA: free fatty acids, IGF-1: insulin-like growth factor-1, IR: insulin resistance, JAK2: Janus kinase 2, M: males, TG: triglyceride.

Multiple publications report the effects of liver-specific impairment in GH signaling, achieved through mutation or deletion of GHR, STAT5, or JAK2 ^[49–51,53,55,56]^. With the exception of the female phenotype in one model ^[55]^, all of these models demonstrate hepatic steatosis, with mechanistic findings including increased hepatic DNL ^[50,56]^, increased hepatic lipid uptake ^[56]^, and decreased hepatic triglyceride secretion ^[50]^. Two of these models also demonstrate hepatic inflammation and hepatocellular injury with increased circulating ALT and AST ^[51,56]^. Experiments to reverse deficits in IGF-1 in these models are instructive with regard to the independent effects of GH versus IGF-1 in the pathogenesis of NAFLD. In their model of liver-specific GHR deletion, Fan and colleagues demonstrate that rescue of GHR expression reverses the liver phenotype, whereas infusion of IGF-1, which lowers serum GH to normal but does not restore hepatic GH signaling, is not sufficient to correct hepatic steatosis ^[50]^. In contrast, in a similar model, Liu et al report that restoration of hepatic IGF-1 expression, which may be more effective at sustaining IGF-1 levels than peripheral infusion, partly but not completely reverses steatosis and normalizes ALT and AST but does not decrease inflammatory cytokines or enzymes involved in oxidative stress ^[56]^. Sos and colleagues illustrate the importance of circulating systemic GH by crossing their mice that have hepatocyte-specific JAK2 deletion with *little* mice that have a GHRH mutation that prevents secretion of GH ^[51]^. In these animals without circulating GH, the fatty liver phenotype is reversed, demonstrating the complexity of these models and the likely importance of GH-driven lipolysis to provide substrate for the development of fatty liver seen in the liver-specific models.

Other crossed models have interrogated additional important aspects of the GH signaling cascade. For example, Themanns et al crossed mice with a hepatocyte-specific deletion of either JAK2 or STAT5 with GH transgenic mice, resulting in two models with very high circulating GH and signaling defects in either JAK2 or STAT5, respectively ^[60]^. Whereas both models showed substantial hepatic steatosis, increased reactive oxygen species (ROS), and eventual hepatocellular malignancy, hepatic JAK2-deficient mice demonstrated later onset of hepatocellular malignancy and reduced oxidative damage from ROS compared to the STAT5 deficient model ^[60]^. The authors attribute the difference in hepatocellular malignancy to activation of STAT3 in mice with STAT5 deficiency and intact JAK2 signaling: STAT3 was not activated in the JAK2 knock-out mice but promoted tumorigenesis in the STAT5 knock-out mice ^[60]^. Additionally, knock-out of JAK2 but not STAT5 resulted in increased expression of glutathione *S*-transferases, which ameliorated oxidative damage despite equally elevated ROS production ^[60]^. This model is described in detail because it illustrates important differences in phenotype depending on where the hepatic GH signaling cascade is impaired, emphasizing the importance of each element in the cascade as well as the effects of potential compensatory changes on metabolism, inflammation, and tumorigenesis.

Two published models report systemic rather than liver-specific impairments in GH signaling. In a model of systemic GHR mutations that impair or inhibit activation of STAT5, Barclay and colleagues demonstrate the development of a NASH phenotype, with steatosis, inflammation, and hepatocellular ballooning, in conjunction with upregulation of genes relating to lipid uptake, transport, and synthesis ^[52]^. Nishizawa and colleagues describe the hepatic phenotype in the spontaneous dwarf rat, which has a mutation in the GH gene resulting in absent circulating GH and substantially reduced circulating IGF-1 ^[54]^. This model also demonstrates development of a NASH phenotype, with steatosis, hepatocellular injury, and fibrosis ^[54]^. In this model, administration of GH or, independently, administration of IGF-1 both reversed the features of NASH ^[54]^.

Taken together, evidence from animal models supports an important, if complex, role for reduced GH and/or IGF-1 in the pathogenesis of NAFLD and NASH. GH signaling in the liver clearly regulates lipid handling, with impaired signaling leading to increased hepatocellular triglyceride accumulation. In the liver-specific knock-out models, steatosis is likely driven at least in part by the substantial rise in circulating FFA that results from high circulating GH. The two models of systemically impaired GH signaling also demonstrate a NAFLD phenotype, however, suggesting a fundamental role for GH in preventing hepatic steatosis. Many models also demonstrate that reduced GH signaling is associated with features of NASH, including inflammation, hepatocellular injury, and fibrosis. It is difficult to determine if these phenotypic features are due to absence of GH signaling, absence of IGF-1 signaling, or lipotoxicity independent of GH or IGF-1. The fact that administration of either GH or IGF-1 ameliorates hepatocellular injury ^[54,56]^ suggests an important role for IGF-1 in the pathogenesis of NASH. IGF-1 did not entirely reverse steatosis or some features of inflammation, however, supporting independent roles for both GH and IGF-1 in the pathogenesis of NAFLD and NASH ^[50,54,56]^.

## 5. Human models: hepatic effects of GH deficiency or resistance

Human models provide further evidence for a role of reduced GH and IGF-1 in the pathogenesis of NAFLD and NASH. Several studies of adolescents and adults with hypopituitarism that includes pituitary GH deficiency (GHD) demonstrate a disproportionately high prevalence of NAFLD compared to controls, often in association with lower serum IGF-1 levels ^[61–65]^. Nishizawa and colleagues recruited 69 adults with hypopituitarism who had GHD and were receiving routine hormonal replacement for all other pituitary axes ^[66]^. Among this group, 77% had NAFLD, compared to a prevalence of 12% in age-, gender-, and BMI-matched controls ^[66]^. Patients with GHD also had higher circulating AST, ALT, triglyceride, and high sensitivity CRP (hsCRP) compared to controls, as well as higher indices of insulin resistance ^[66]^. Sixteen patients who had GHD, NAFLD by ultrasound, and laboratory evidence of liver dysfunction underwent liver biopsy; of this group, fourteen had evidence of NASH ^[66]^. Five of these patients with NASH received GH replacement therapy for 6 to 12 months followed by repeat liver biopsy and biochemical assessment; GH therapy significantly improved steatosis and fibrosis compared to pre-treatment histology and reduced ALT and ALT ^[66]^. Matsumoto and colleagues report a similar effect of GH replacement to reduce ALT and AST in a retrospective study of adults with GHD comparing those who received GH replacement with a group that did not ^[67]^. After 24 months, ALT and AST were significantly lower in those receiving GH compared to the control group; sustained reductions in ALT and AST over 24 months were also reported in a subgroup of 13 individuals with GHD and NASH who were treated with GH ^[67]^. Similarly, humans with Laron syndrome, characterized by GH resistance, exhibit NAFLD at a relatively high prevalence of 6 out of 11 adults studied ^[68]^.

Acromegaly, a state of GH excess, also provides a useful model of the effects of GH on hepatic lipid. In cross-sectional analyses comparing individuals with acromegaly to matched controls, those with acromegaly have lower hepatic lipid content ^[69,70]^. Further, following treatment of acromegaly with transsphenoidal pituitary surgery and/or medical therapy, intrahepatic lipid content increases ^[69,71,72]^. Interestingly, reduced hepatic lipid occurs in acromegaly despite the significant insulin resistance that characterizes the acromegalic state and is expected to promote NAFLD. Using ^31^P/^1^H-magnetic resonance spectroscopy (MRS) to evaluate hepatic mitochondrial function (^31^P-MRS) and lipid content (^1^H-MRS) in individuals with acromegaly and matched controls, Fellinger and colleagues suggest that this “disconnect” between insulin resistance and hepatic lipid content may be secondary to increased hepatic mitochondrial activity in those with acromegaly, who demonstrated 50% higher ATP synthesis rate compared with controls ^[70]^. Thus, despite the insulin resistance associated with increased GH, the GH-associated increase in mitochondrial function and lipid beta-oxidation results in a net reduction in intrahepatic lipid in acromegaly.

## 6. The GH/IGF-1 axis in individuals with NAFLD/NASH

Research increasingly demonstrates that individuals with NAFLD/NASH demonstrate reductions in GH and/or IGF-1 in association with more severe disease. Multiple factors may be at play in explaining the data described below. First, NAFLD and NASH occur most commonly in individuals with obesity, particularly abdominal or visceral obesity; these individuals are known, on average, to have relative reductions in GH secretion ^[9,10]^. Thus, in simple steatosis and early NASH, many of the body composition and metabolic features predisposing to NAFLD/NASH may also contribute to reduced GH and/or IGF-1. Second, with the significant hepatic dysfunction common to the later stages of many liver diseases, the GH signaling cascade is impaired as is synthesis of IGF-1 by hepatocytes ^[73]^. Finally, through mechanisms not yet completely elucidated, cirrhosis is associated with a state of GH resistance, with high circulating GH and low circulating IGF-1 ^[74]^. With these considerations, individuals with NAFLD/NASH may have variable changes in the GH/IGF-1 axis depending on the severity of their disease. The discussion below primarily concerns individuals with simple steatosis, NASH, and/or relatively earlier stages of fibrosis, not those with cirrhosis and end-stage liver disease.

GH secretory capacity, measured by peak GH levels in response to stimulation testing, is generally reported as lower in individuals with NAFLD compared to controls ^[75,76]^. For example, in a cross-sectional study of 102 adults who underwent provocative GH testing with arginine and GHRH, mean peak stimulated GH was lower in those with NAFLD compared to controls after adjustment for age, sex, visceral adipose tissue, and fasting glucose ^[76]^. Higher peak GH was associated with lower serum ALT levels, and overall peak GH predicted 14.6% of the variability in intrahepatic lipid in adjusted models ^[76]^. Circulating IGF-1 levels are consistently reported as lower in individuals with NAFLD compared to matched controls ^[75–79]^, and IGF-1 levels are further reduced at advanced stages of steatohepatitis and fibrosis compared with levels in those with simple steatosis ^[45,78,80–82]^. Like serum levels, hepatic IGF-1 mRNA levels also decline with increasing degrees of steatosis, steatohepatitis, and fibrosis in adults ^[28,83]^, whereas IGF-1R and GHR expression does not appear to change with increasing severity of NAFLD ^[28]^. Given that the liver is the primary source of circulating IGF-1, the relationship between increasing NAFLD severity and lower IGF-1 levels may be bidirectional. That is, lower IGF-1 could be causal in the pathogenesis of NASH, and increasing levels of hepatic impairment, in turn, cause reduced IGF-1 synthesis. In contrast, impaired liver function would be expected to cause GH resistance rather than reduced GH, supporting the possibility that reduced GH secretory capacity found in individuals with NAFLD may contribute to NAFLD onset and progression.

## 7. Modulating the GH axis to treat NAFLD

Recent clinical trials provide important causal evidence regarding the role of reduced GH and IGF-1 in the pathogenesis of NAFLD. At present, much of this work comes from studies in individuals with HIV-infection, many of whom have a phenotype of increased visceral adiposity (for multiple reasons beyond the scope of this discussion) and high risk for NAFLD. A GHRH analog, tesamorelin, is used to reduce visceral fat in this population ^[84,85]^, and our group has conducted two randomized controlled trials (RCTs) investigating the effects of tesamorelin on hepatic lipid content. In the first, individuals with HIV-infection and obesity who were not selected for NAFLD received tesamorelin vs placebo for six months, after which the tesamorelin group demonstrated a reduction in hepatic lipid content compared with placebo ^[86]^. In a subsequent RCT of individuals with HIV-infection and NAFLD, tesamorelin treatment over 1 year compared with placebo achieved a 37% relative reduction in hepatic lipid content and also prevented progression of fibrosis ^[87]^. Secondary mechanistic studies among this cohort have demonstrated that tesamorelin upregulated gene pathways in the liver related to oxidative phosphorylation and downregulated pathways related to inflammation ^[30]^. Further, tesamorelin reduced circulating proteins related to cytotoxic T-cell and monocyte activation in conjunction with downregulation of hepatic gene pathways related to immune activation ^[88]^. An ongoing study is investigating the effects of tesamorelin in individuals with NAFLD who do not have HIV-infection. Among the general population, Bredella and colleagues demonstrated reductions in intrahepatic lipid among men with abdominal obesity, not selected for NAFLD, who received 6 months of recombinant human GH (rhGH) compared with those receiving placebo ^[89]^. In a pilot study among young adults with NAFLD, our group demonstrated that physiologic GH therapy for 6 months tended to reduce hepatic lipid, with relative reduction of 36% (95% CI, −88%, 16%) in hepatic fat fraction, although these results did not achieve statistical significance ^[90]^. More recently, Dichtel, Miller, and colleagues have completed a larger randomized trial of rhGH in 52 adults with NAFLD. In this study, rhGH therapy was effective in reducing liver fat and ALT, without worsening glycemia ^[91]^. It is important to note that GH excess may have multiple adverse effects including insulin resistance and potential promotion of neoplastic growth. Augmentation of GH to address the pathophysiology of NAFLD/NASH likely is most appropriate to restore normal GH physiology, for example, in individuals with relative GH deficiency associated with abdominal obesity. Increasing GH to supraphysiological levels would incur multiple risks, and GH augmentation may not be appropriate for all individuals with NAFLD/NASH. Additionally, exercise and weight loss are known to augment GH secretion. We are not aware of published interventional studies elaborating links between exercise and/or weight loss, altered GH secretory dynamics, and changes in NAFLD, and such studies would be of substantial interest to determine if increased GH secretion is one mechanism whereby exercise and weight loss improve hepatic steatosis. Other GH secretagogues, including GH-releasing peptides (GHRPs) and selective ghrelin receptor agonists, also require further study in NAFLD.

## 8. Summary and future directions

Abundant evidence suggests that impairment in the GH/IGF-1 axis may contribute to the pathogenesis of NAFLD and NASH. GH signaling in the liver reduces lipid storage by suppressing DNL and promoting lipid beta-oxidation, and animal and human studies also support these key functions of GH. Further, GH and IGF-1 have anti-inflammatory effects, and IGF-1 exerts an anti-fibrogenic effect, actions that are also borne out in animal and human models. Augmenting GH (and, consequently, IGF-1) with the GHRH analog tesamorelin leads to upregulation of gene pathways involved in beta-oxidation and also has a substantial anti-inflammatory effect, downregulating pathways associated with inflammation and immune activation and reducing serum concentration of proteins associated with T-cell and monocyte activation. There currently are no medications with specific regulatory approval to treat NAFLD/NASH ^[92]^, despite multiple past and current trials focusing on various pathways in pathogenesis. Given the evidence supporting a role for reduced GH/IGF-1 in pathogenesis, further study is needed to investigate whether strategies to augment GH/IGF-1 are safe and effective for some individuals with NASH. Such strategies may include use of a GHRH analog, use of rhGH, and/or other methods to increase GH and/or IGF-1, including other GH secretagogues. Importantly, just as there are multiple paths to developing NAFLD/NASH, multiple treatment strategies will likely be needed, and augmenting GH/IGF-1 may not be an effective strategy for all individuals. Given the association between abdominal obesity and reduced GH secretion, however, augmentation of GH may be one effective strategy to address NAFLD/NASH in those with NAFLD who also exhibit obesity or excess abdominal adiposity.

## Author contributions

I.L.M. and T.L.S. wrote the manuscript, and T.L.S. takes primary responsibility for the final content. I.L.M. and T.L.S. have read and approved the final manuscript.

## Conflicts of interest

I.L.M. has no conflicts to disclose. T.L.S. has received funding to her institution from Pfizer, Inc., unrelated to this work.

## Funding

This work is partially supported by NIH grants R01DK114144 (Growth Hormone Releasing Hormone Analog to Improve Nonalcoholic Fatty Liver Disease and Associated Cardiovascular Risk to T.L.S.) and P30DK040561 (Nutrition Obesity Research Center at Harvard).
